# Senescent renal tubular epithelial cells activate fibroblasts by secreting Shh to promote the progression of diabetic kidney disease

**DOI:** 10.3389/fmed.2022.1018298

**Published:** 2023-01-25

**Authors:** Dan Wang, Ling Yin, Rongyu Chen, Wanlin Tan, Luqun Liang, Jiayi Xiang, Huifang Zhang, Xingcheng Zhou, Huaqing Deng, Bing Guo, Yuanyuan Wang

**Affiliations:** ^1^Department of Pathophysiology, School of Basic Medicine, Guizhou Medical University, Guiyang, China; ^2^Guizhou Provincial Key Laboratory of Pathogenesis and Drug Research on Common Chronic Diseases, Guizhou Medical University, Guiyang, Guizhou, China; ^3^International Scientific and Technological Cooperation Base of Pathogenesis and Drug Research on Common Major Diseases, Guizhou Medical University, Guiyang, China; ^4^Division of Nephrology, Jiangsu Provincial Hospital of Chinese Medicine, Nanjing, Jiangsu, China; ^5^Guizhou Province Innovation Base of Common Major Chronic Disease Pathogenesis and Drug Development and Application, Guiyang, Guizhou, China; ^6^Key Laboratory of Endemic and Ethnic Diseases, Ministry of Education, Guizhou Medical University, Guiyang, Guizhou, China; ^7^Department of Pathophysiology, Guizhou Medical University, Guiyang, Guizhou, China

**Keywords:** diabetic kidney disease, renal tubular epithelial cells, senescence, Shh, inflammation, SASP

## Abstract

**Introduction:**

Diabetic kidney disease (DKD) is one of the complications of diabetes; however, the pathogenesis is not yet clear. A recent study has shown that senescence is associated with the course of DKD. In the present study, we explored whether senescent renal tubular cells promote renal tubulointerstitial fibrosis by secreting Sonic hedgehog (Shh) which mediates fibroblast activation and proliferation in DKD.

**Methods:**

A 36-week-old *db/db* mice model and the renal tubular epithelial cells were cultured in high glucose (HG, 60 mmol/L) medium for *in vivo* and *in vitro* experiments.

**Results:**

Compared to *db/m* mice, blood glucose, microalbuminuria, serum creatinine, urea nitrogen, and UACR (microalbuminuria/urine creatinine) were markedly increased in *db/db* mice. Collagen III, monocyte chemoattractant protein-1 (MCP-1), and tumor necrosis factor-alpha (TNF-α) were also increased in *db/db* mice kidneys, suggesting fibrosis and inflammation in the organ. Moreover, the detection of SA-β-galactosidase (SA-β-Gal) showed that the activity of SA-β-Gal in the cytoplasm of renal tubular epithelial cells increased, and the cell cycle inhibition of the expression of senescence-related gene cell cycle inhibitor p16^*INK*4*A*^ protein and p21 protein increased, indicating that renal fibrosis in *db/db* mice was accompanied by cell senescence. Furthermore, Shh is highly expressed in the injured renal tubules and in the kidney tissue of *db/db* mice, as detected by enzyme-linked immunosorbent assay (ELISA). The results of immunofluorescence staining showed increased positive staining for Shh in renal tubular epithelial cells of *db/db* mice and decreased positive staining for Lamin B1, but increased positive staining for γH2A.X in cells with high Shh expression; similar results were obtained *in vitro*. In addition, HG stimulated renal tubular epithelial cells to secrete Shh in the supernatant of the medium. D-gal treatment of renal tubular epithelial cells increased the protein levels of Shh and p21. We also found enhanced activation and proliferation of fibroblasts cultured with the supernatant of renal tubular epithelial cells stimulated by HG medium but the proliferative effect was significantly diminished when co-cultured with cyclopamine (CPN), an inhibitor of the Shh pathway.

**Discussion:**

In conclusion, HG induces renal tubular epithelial cell senescence, and the secretion of senescence-associated proteins and Shh mediates inflammatory responses and fibroblast activation and proliferation, ultimately leading to renal fibrosis.

## 1. Introduction

Diabetes mellitus (DM) is a chronic metabolic disease characterized by hyperglycemia, which affects the level of energy metabolism disorder, leading to various organ lesions and complications. The incidence of DM has increased dramatically in the last 30 years, threatening human health (1). Complications occur in patients with diabetes, among which the microvascular complications are known as diabetic kidney disease (DKD). It eventually progresses to end-stage renal disease (ESRD), reducing the long-term survival rate of patients (2). The pathogenesis of DKD is complex and unclear. Current studies have suggested that genetic factors, senescence, autophagy, metabolism impairment, and other pathogenic factors are associated with the DKD. Fu et al. reported that stress-induced senescence of renal tubular epithelial cells is a major biological event in DKD renal tubular cells (3).

Luo et al. revealed that among the adverse factors of external stimuli, renal tubular epithelial cells experienced senescence and accelerated kidney fibrosis through Wnt9a signaling pathway. The study also found that the expression of senescence-related cell cycle inhibitors, p16^*INK*4*A*^ and p21 proteins, was upregulated (4). Kitada et al. (5) showed that a high concentration of glucose induced senescence of renal tubular epithelial cells through the p21 pathway and the activation of an inflammatory response, and rapamycin target protein (mTOR) signaling, which in turn promoted renal tubular epithelial cell fibrosis. During this process, senescent cells secrete cytokines, chemokines, extracellular matrix proteins, and growth factors, which are secretion phenotypes related to senescence, and all secreted cytokines are defined as senescence-associated secretory phenotypes (SASPs) (6). SASP is the cause of sustained senescence and detrimental effects on cells that could lead to chronic inflammation and fibrosis (7). Many studies have shown that senescent cells aggravate kidney pro-fibrotic milieu through SASP. In mouse models and patients with DKD, senescence of renal tubular epithelial cells aggravates kidney fibrosis (8).

Sonic hedgehog (Shh) is a secretory protein that is indispensable in the growth and development of many organs in mammals (9). In human osteoarthritis, Shh mediates the growth and senescence of mesenchymal stem cells. Previous studies (10, 11) also showed that Shh was upregulated in chronic lung and liver diseases, and in the process of biliary fibrosis, activating Hh signaling in immature ductular cells promoted epithelial-mesenchymal transition (EMT). *In vivo* and *in vitro* studies by Kramann et al. demonstrated that inhibition of GLI1 might be a therapeutic strategy for limiting the proliferation of fibroblasts in renal fibrosis. These studies indicated that Shh signal transduction pathway is related to renal development and fibrosis (12, 13). However, the mechanism of senescent renal tubular cells involved in tubulointerstitial fibrosis during DKD remains unclear. In the present study, we found that senescent renal tubular epithelial cells promote activation and proliferation of fibroblasts by secreting Shh, thereby accelerating DKD progression.

## 2. Materials and methods

### 2.1. Chemicals and antibodies

Chemicals and antibodies were as follows: anti-fibronectin (FN) (Proteintech, China), anti-Collagen III (Col-III) (Proteintech, China), anti-Shh (Proteintech, China), anti-tumor necrosis factor-alpha (TNF-α) antibody (Santa Cruz, USA), proliferating cell nuclear antigen (PCNA) (Proteintech, China), MCP-1 polyclonal antibody (Proteintech, China), anti-GAPDH antibody (Wuhampmack Biotechnology Co., Ltd., China), Vimentin polyclonal antibody (Proteintech, China), anti-p21 (Santa Cruz, USA), anti-p16^*INK*4*A*^ (Santa Cruz, USA), anti-platelet-derived growth factor receptor β (PDGFR-β) (Santa Cruz, USA), Lamin B1 (Proteintech, China), and γH2A.X polyclonal antibody (Proteintech, China); hypersensitive ECL chemiluminescence kit (Beyotime Biotechnology, China), creatinine, triglyceride, total cholesterol (T-CHO) test kit (Nanjing Jiancheng Bioengineering Institute, China), and microalbumin test kit (Elabscience Biotechnology Co., Ltd., China); H&E staining kit and sirius red staining kit (Solarbio, China); SA-β-galactosidase (SA-β-Gal) staining kit (Solarbio, China), DMEM cell culture medium (DMEM; Gibco, CA, USA); blood glucose (BG) meter and BG test paper (Johnson Co., Ltd., USA); PVDF membrane and Whatman filter paper (Millipore, USA), BCA protein concentration assay kit (Beyotime Biotechnology, China), Shh enzyme-linked immunosorbent assay (ELISA) detection kit (Jianglai Biology, China) and cell cycle detection kit (KGI Bio Co., China), Cyclopamine (CPN) (MedChemExpress, USA), D-galactose (D-gal) (Solarbio, China).

### 2.2. Animal models

Clean grade 8-week-old *db/db* mice, weighing 50 ± 20 g, were purchased from the Institute of Model Animals, Nanjing University {the batch number: [SCXK (Su) 2018-0008]}.

The animal models were replicated, randomly divided into *db/m* mice and *db/db* mice (*n* = 5 per group), and were raised in the Experimental Animal Center of Guizhou Medical University. All groups were fed aseptic feed and allowed to drink *ad libitum*. The mice were fed from 8 weeks and euthanized after 36 weeks. Before euthanasia, urine was collected, and mice were fasted but not hydrated for 6–8 h. Also, the serum was maintained overnight at 4°C, followed by centrifugation; the serum was stored at −80°C. After the mice were euthanized, 5 ml physiological saline was injected into the left atrial appendage to flush the organs. A part of the kidneys was removed and fixed in 3.7% paraformaldehyde, while the other part was stored at –80°C. BG, microalbumin (MUA), triglyceride (TG), T-CHO, serum creatinine (SCr), blood urea nitrogen (BUN), urine creatinine (UCr), and Urinary Albumin Creatinine Ratio (UACR) (MUA/UCr) were analyzed following the kit’s instructions.

### 2.3. Cell culture

NRK-52E and NRK-49F cells were obtained from Jennio Biotech (Guangzhou, China) and cultured in a cell incubator. The experiments were grouped as follows: Normal glucose (NG) group: 2% fetal bovine serum (FBS) normal glucose (5.5 mmol/L); High glucose (HG) group: 2% FBS high glucose (60 mmol/L). The cells in the logarithmic growth phase were digested with trypsin and inoculated in a 6-well plate for culture. After the density reached about 60%, NEK-52E cells were starved for 12 h and switched to HG medium for 72 h. The NG and HG supernatants of NRK-52E cells cultured for 3–8 generations in 6-well plates were collected, and the content of Shh in the supernatant was detected by ELISA. After NRK-49F cell density reached about 60%, the medium in the 6-well plate was replaced with the supernatant of the previously collected NG and HG condition medium of NRK-52E cells; the cell protein was collected after 72-h culture. Immunofluorescence staining and protein collection of NRK-52E cells treated with 4 mg/ml D-gal for 72 h. NRK-49F cells were cultured with 5 and 10 nM Shh inhibitor cyclopamine (CPN) in condition medium for 72 h, and the total protein was collected.

### 2.4. Pathological examination and immunohistochemical staining of renal tissue

The renal tissue specimens of mice were subjected to dehydration, embedding, and paraffin sectioning. Then, the sections were dyed with hematoxylin-eosin and sirius red reagents following the recommended specifications. The distribution and expression of p16^*INK*4*A*^ in kidney tissues of mice in each group were detected by immunohistochemical staining. Paraffin sections were dewaxed and hydrated and, incubated with 3% hydrogen peroxide deionized water, followed by antigen retrieval by microwave. Anti-Shh (1:50, Santa Cruz, USA) and Anti-p16^*INK*4*A*^ (1:100, Santa Cruz, USA) were used a protein antibodies at 4°C. Subsequently, DAB kit (ZSGB-BIO, Beijing, China) was used for immunohistochemical staining. The pathological changes of the renal tissue were examined. Percentage of inflammatory cells, denatured cells, and collagen deposition in the entire region of the renal tubules (14). The positive staining area was quantified by ImageJ software (NIH, USA), six random fields per sample).

### 2.5. Immunofluorescence staining

After resection and isolation from mice, kidney tissues were immediately frozen in liquid nitrogen, embedded into optimal cutting temperature (OCT) compound, and sectioned into 3-μm-thick slices. The frozen sections were fixed in 3.7% paraformaldehyde for 10 min, washed with 0.8% Tris Buffered Saline with Tween-20 (TBST) (TBS with Tween-20) permeabilized with 0.5% Triton X-100 for 5 min, blocked with 10% donkey serum for 1 h. Subsequently, the sections were incubated with the primary antibody for 16 h, and incubated secondary antibody, followed by treatment with anti-quenchable sealing tablets containing 4′,6-diamidino2-phenylindole (DAPI). The slides were sealed with coverslips, and the sections were observed under a fluorescence microscope. The primary antibodies used for immunohistochemical staining were anti-Col-III (1:200, Proteintech, China), anti-Lamin B1 (1:50, Proteintech, China), anti-Shh (1:50, Santa Cruz, USA), and γ H2A.X polyclonal antibody (1:100, Proteintech, China). The secondary antibody was labeled with fluorescein isothiocyanate (FITC) or CY3 (1: 200, Santa Cruz, USA). The positive staining area was quantified by ImageJ software, and six random fields per sample were examined.

### 2.6. SA-β-galactosidase assay kit

The SA-β-Gal activity of renal frozen sections and cells was stained with SA-β-Gal staining kit (Solarbio, China) to detect cell senescence. The procedure was in accordance with the reagent instructions.

### 2.7. Western blotting

The renal tissue and cells are lysed on ice with lysis buffer, and protein concentration was determined. The samples were separated by 12% sodium dodecyl sulfate polyacrylamide gel electrophoresis (SDS-PAGE) and electrotransferred to PVDF membrane (Microporous, USA). Subsequently, the membrane was blocked for 2 h, and probed with primary antibody at 4°C, such as rabbit-anti-fibronectin (1:1,000), rabbit-anti-Shh (1:1,000; Proteintech), rabbit-anti-Shh (1:500; Santa Cruz), rabbit-anti-TNF-α (1:1,000; Santa Cruz), rabbit-anti-MCP-1 (1:1,000, Proteintech), rabbit-anti-PCNA (1:1,000; Proteintech), rabbit-anti-Vimentin (1:1,000; Proteintech), mouse-anti-p21 (1:1,000; Santa Cruz), mouse-anti-p16^*INK*4*A*^ (1:1,000; Santa Cruz), rabbit-anti-PDGFR-β (1:1,000; Santa Cruz), rabbit-anti-β-actin (1:8,000; Proteintech), and anti-GAPDH antibody (1:5,000; Wuhampmack Biotechnology Co., Ltd., China) for 16 h at 4°C. The chemiluminescence signal was detected after incubation with second antibody for 1 h. Finally, ImageJ software were used for analysis.

### 2.8. Flow cytometry

The apoptotic rate of NRK-52E cells was detected using an cell cycle detection kit. The cells were grouped into two NG and HG groups. Approximately, 1 × 10^5^ cells are collected and fixed with 70% ethanol at 4°C for 16 h, followed by staining with 500 μL PI/RNase a solution at room temperature in the dark for 60 min. Finally, the cell cycle was detected by flow cytometry at the excitation/emission wavelength of 488 nm. The data were analyzed by ACEA NovoCyte software (China), and the proportion of the G1, S, and M phase of NRK-52E cells cultured in NG and HG was obtained, respectively. The procedure was in accordance with the manufacturer’s instructions.

### 2.9. ELISA

Sonic hedgehog content in the cell culture medium was detected using ELISA kits (Jianglai Biology, JL30789). The procedure was in accordance with the reagent instructions.

### 2.10. Statistical analysis

All experiments were conducted in at least three biological replicates. The comparison between two groups was made using Student’s *t*-test. Data were expressed as mean ± standard deviation (SD). SPSS 23.0 software (IBM, USA) was used for statistical analysis. *P* < 0.05 indicated a statistically significant difference.

## 3. Results

### 3.1. Biochemical characteristics and histopathological alterations of diabetic mice

**FIGURE 1:**
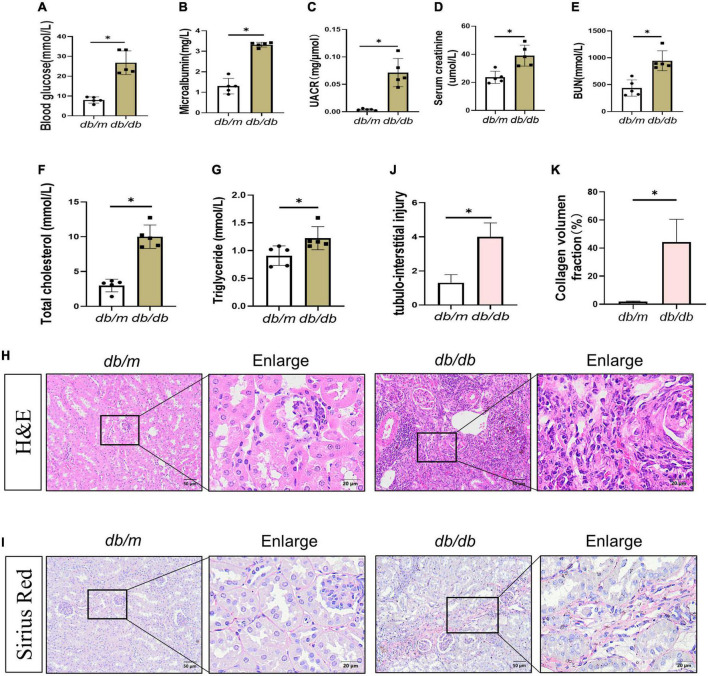
Changes in biochemical indexes and renal morphology in *db/db* mice. **(A–E)** Blood glucose **(A)**, microalbumin **(B)**, UACR **(C)**, serum creatinine **(D)**, blood urea nitrogen **(E)**, total cholesterol **(F)**, and triglyceride **(G)** levels were increased. H&E staining **(H)** showed kidney histologic changes in *db/db* mice and *db/m* mice at 36 weeks and to assess the tubular injury index **(J)**. Bar = 50 μm. Boxed areas are enlarged. Bar = 20 μm. **(I,K)** Sirius red staining (SR) showed collagen deposition in *db/db* mice kidneys at 36 weeks **(I)**, collagen deposition fraction **(K)**. Boxed areas are enlarged. Bar = 20 μm. All data are presented as mean ± standard deviation (SD) from three independent experiments. *n* = 5; *P < 0.05 vs. *db/m* group.

Microalbuminuria (MUA) is one of the earliest clinical evidence of DKD (15, 16). The ratio of BG, MUA, UACR, SCr, BUN, T-CHO, and TG levels increased in *db/db* mice (Figures 1A–G). According to the diagnostic guidelines of DKD, renal pathological changes are the major diagnostic criteria. The main pathological changes are mesangial matrix dilatation, glomerular basement membrane thickening, and the presence of nodular disease (17). Recent findings supported that proximal tubular and tubulointerstitial injury is a critical part of DKD and interacts with interstitial fibrosis to form a vicious cycle that promotes the pathogenesis of renal fibrosis (18, 19). Therefore, we evaluated the histopathological alterations in kidneys of DKD. Hematoxylin and eosin (H&E) staining showed that *db/m* mice had complete glomerular morphology, while *db/db* mice had partial glomerular expansion, mesangial cell proliferation, and basement membrane thickening. Conversely, focal segmental atrophy of the tubules and granular degeneration of some tubular epithelial cells with focal lymphocytic infiltration and extracellular matrix (ECM) deposition in the renal interstitium were observed in the kidneys of *db/db* mice (Figures 1H, J). In addition, sirius red staining showed that the renal tissue of *db/m* mice was structurally intact with almost no deposition of sirius red-positive staining. In contrast, the kidney tissue structure of *db/db* mice was disturbed, and sirius red-positive staining in the renal interstitium was significantly increased compared to *db/m* mice (Figures 1I, K), indicating an increased collagen deposition. These data suggested the occurrence of kidney damage and fibrosis in *db/db* mice.

### 3.2. Increased senescence, fibrosis, and Shh in the kidney of *db/db* mice

**FIGURE 2:**
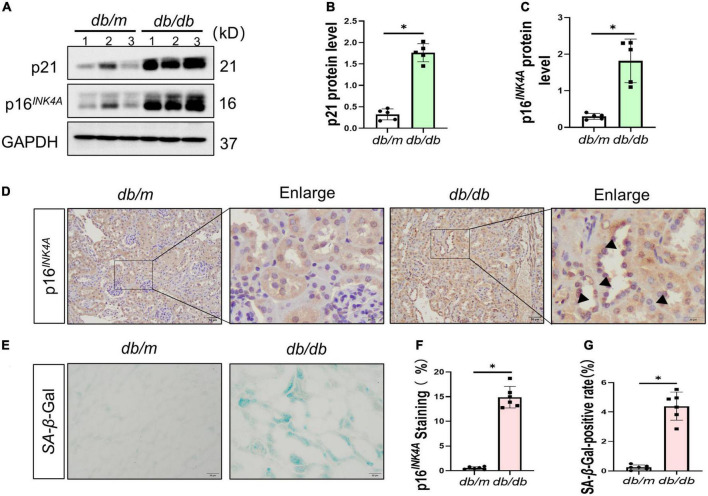
Senescence in the process of diabetic kidney disease (DKD). Western blot analyses showed kidney p21 and p16^*INK*4*A*^ protein in renal tissues. Representative Western blot **(A)** and quantitative data **(B,C)** are presented. **(D,F)** Immunohistochemical staining showed p16^*INK*4*A*^ expression in *db/db* mice and *db/m* mice diseased kidneys. Black arrows indicate positive staining in tubules. Boxed areas are enlarged. Bar = 20 μm. **(E,G)** Detection of SA-β-galactosidase (SA-β-Gal) activity in renal tissue. **(E)** Representative images of renal tissues are shown. Scale bar = 50 μm. The ratio of SA-β-Gal-positive cells is presented in the panel **(G)**. All data are presented as mean ± standard deviation (SD) from three independent experiments. *n* = 5; *P < 0.05 vs. *db/m* group.

**FIGURE 3:**
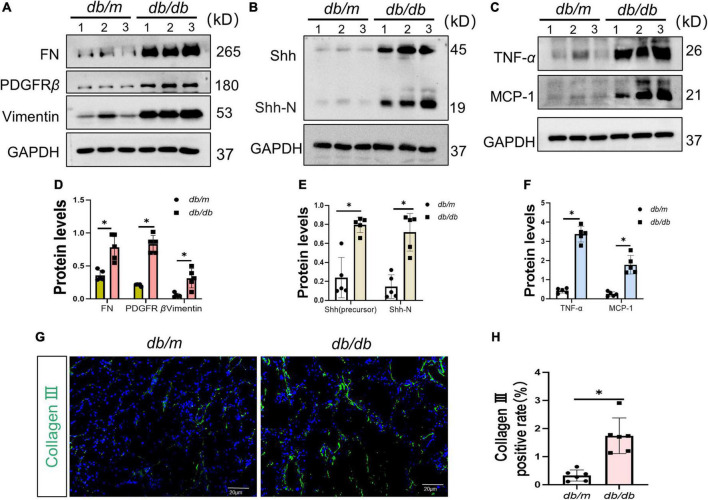
Fibrosis and senescence-associated secretory phenotype (SASP) in the process of diabetic kidney disease (DKD). **(A,D)** Western blot analyses showed FN, PDGFR-β, and Vimentin protein in renal tissues. Representative Western blot **(A)** and quantitative data **(D)** are presented. **(B,C,E,F)** Western blot analyses showed Sonic hedgehog (Shh) (precursor), Shh-N, tumor necrosis factor-alpha (TNF-α), and MCP-1 protein in renal tissues. Representative Western blot **(B,C)** and quantitative data **(E,F)** are presented. **(G,H)** Representative micrographs showed immunofluorescence staining of Collagen-III in different groups **(G)**, and quantitative analysis **(H)**. DAPI staining (blue) represents cell nucleus, Bar = 20 μm. All data are presented as mean ± standard deviation (SD) from three independent experiments. *n* = 5; *P < 0.05 vs. *db/m* group.

**FIGURE 4:**
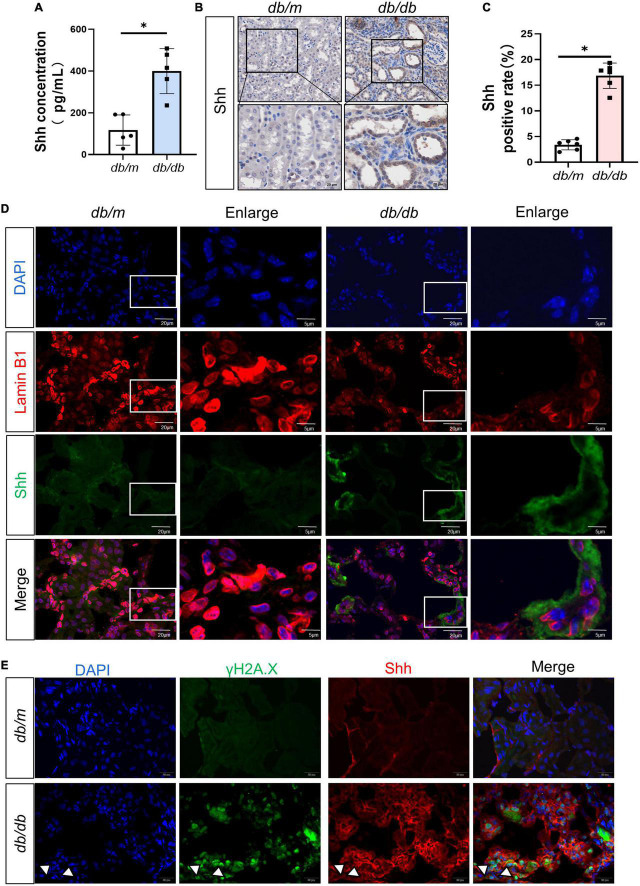
Expression and distribution of Sonic hedgehog (Shh) in the process of diabetic kidney disease (DKD). **(A)** The content of Shh in renal tissue was detected by enzyme-linked immunosorbent assay (ELISA). **(B,C)** Representative micrographs show immunohistochemical staining of Shh in different groups **(B)** and quantitative analysis **(C)** of Shh. **(D)** Representative micrographs showed immunofluorescence staining of Lamin B1 and Shh in different groups. DAPI staining (blue) represents cell nucleus, Bar = 20 μm. The enlarge images indicate the co-expression of positive staining cells. Bar = 5 μm. **(E)** Representative micrographs showed immunofluorescence staining of γH2A.X and Shh in different groups. White arrows indicate the co-expression of positive staining cells. DAPI staining (blue) represents cell nucleus, Bar = 20 μm. All data are presented as mean ± standard deviation (SD) from three independent experiments. *n* = 5; *P < 0.05 vs. *db/m* group.

To ascertain the expression of senescence genes in *db/db* mice, we systematically examined the expression of senescence-specific markers p16^*INK*4*A*^ and p21 in the kidney. Typically, the characteristics of aging cells are increased levels of p16^*INK*4*A*^ protein and p21 (20). First, the expression of p16^*INK*4*A*^ and p21 protein elevated in *db/db* mice (Figures 2A–C). The immunohistochemical staining further revealed an increased expression of p16^*INK*4*A*^ in *db/db* mice compared to *db/m* mice (Figures 2D, F). As a marker of senescence, the activity of senescence-associated beta-galactosidase (SA-β-Gal) enhanced during senescence (21) (Figures 2E, G). Moreover, Western blots demonstrated a consistent increase in FN, PDGFRβ, Vimentin, MCP-1, TNF-α, and Shh protein levels in whole kidney lysates of *db/db* mice (Figures 3A–F). In addition, protein encoded by *SHH* gene form precursor and is automatically processed into an amino-terminal domain and a carboxyl-terminal domain (22). Hedgehog precursor protein needs to be modified by N-terminal palmitoylation of hedgehog acyltransferase before Shh can function as a signaling molecule (23). According to the Shh antibody, the molecular weight of Shh precursor, amino-terminal peptide, and molecular weight of Shh carboxy-terminal peptide is 45, 19, and 27 kDa, respectively. As shown in Figure 3B, Shh precursors and Shh amino terminal (Shh-N) peptides are markedly increased in *db/db* mice compared to *db/m* mice. Moreover, the typical manifestation of renal fibrosis is increased expression and deposition of ECM proteins. Collagen type I and type III are two subtypes of the collagen superfamily, which were the major components of interstitial ECM in fibrotic tissues (24). Next, we examined the Col-III fluorescence areas in kidney tissue of *db/db* mice significantly increased by immunofluorescence staining (Figures 3G, H). Compared to *db/m* mice, the content of Shh increased obviously in the kidney tissue of *db/db* mice (Figure 4A). The immunohistochemistry staining provided similar results (Figures 4B, C). Another study has shown found that a decrease of Lamin B1 in senescent cells is a new marker of cellular senescence (25). Interestingly, immunofluorescence staining showed that Lamin B1 red fluorescence reduced in *db/db* mouse kidney tissue, green fluorescence for Shh-positive staining increased significantly, and enhanced green fluorescence of Shh positive staining was observed in the cells of reduced Lamin B1 red fluorescence (Figure 4D). During senescence, one of the characteristics of the cells is the occurrence of macromolecular injury, including DNA damage. γH2A.X belongs to the histone H2A family, which is synthesized in G1 and S phases. It is involved in nucleosomal organization of chromatin together with other histone protein, and is a biomarker of cellular response to DNA double strand breaks and could clearly reflect the status of DNA damage and repair (26, 27). The *in vivo* immunofluorescence results showed that the expression of γH2A.X increased in the cells with high Shh expression (Figure 4E). These findings indicated that *db/db* mice had severe renal senescence and fibrosis, and Shh expression increased in senescent renal tubular cells.

### 3.3. High glucose aggravated the senescence and fibrosis of renal tubular epithelial cells and Shh upregulated in senescent renal tubular epithelial cells

**FIGURE 5:**
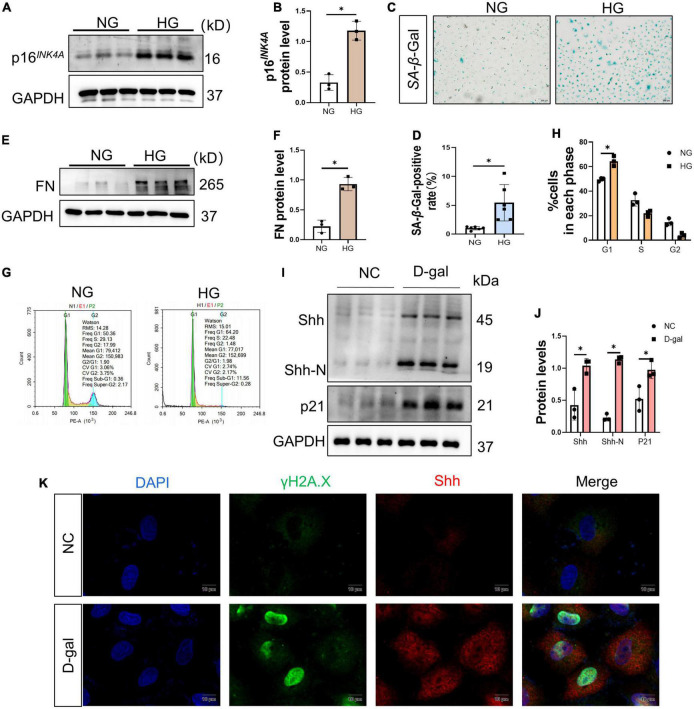
High glucose (HG) mediates renal tubular epithelial cell senescence and fibrosis. **(A,B)** Western blot analyses showed p16^*INK*4*A*^ protein in each cell group. Representative Western blot **(A)** and quantitative data **(B)** are presented. **(C,D)** Detection of SA-β-galactosidase (SA-β-Gal) activity in renal tubular epithelial cells. **(C)** Representative images of renal tubular epithelial cells are shown. The ratio of SA-β-Gal-positive cells is presented in the panel **(D)**. Scale bar = 200 μm. **(E,F)** Western blot analyses showed the FN protein level in each cell group. Representative Western blot **(E)** and quantitative data **(F)** are presented. **(G,H)** Flow cytometry analysis of each group of cell cycle **(G)** and quantitative data **(H)**. HG prevents cell exit from G1 into S phase. Represents the high percentage of G1 phase in the HG group than in the NG group. **(I,J)** Western blot analyses showed Sonic hedgehog (Shh) and p21 protein in each cell group. Representative Western blot **(I)** and quantitative data **(J)** are presented. **(K)** 4 mg/ml D-gal intervention in NRK-52E cells. Representative micrographs showed immunofluorescence staining of γH2A.X and Shh in different groups. DAPI staining (blue) represents cell nucleus, Bar = 10 μm. All data are presented as mean ± standard deviation (SD) from three independent experiments. *n* = 3; *P < 0.05 vs. NC group.

Then, the potential effects of HG on renal tubular epithelial cells were further investigated adopting the *in vitro* model system. Thus, NRK-52E cells were cultured with HG for 72 h. Consistent with the data *in vivo*, p16^*INK*4*A*^ protein increased in the HG group (Figures 5A, B) and the activity of SA-β-Gal increased in the HG group (Figures 5C, D). In addition, that the level of FN protein enhanced in the HG group compared to the NG group (Figures 5E, F). A large number of cells in the HG group stagnated in the G1 phase (Figures 5G, H). The above findings were corroborated by another experiment. Herein, we used 4 mg/ml of D-gal to induce senescence in renal tubular epithelial cells and found that it upregulated the levels of p21 protein, Shh precursor, and the Shh-N peptide (Figures 5I, J). Simultaneously, the increased expression of γH2A.X and Shh in the cells, and in cells where γH2A.X was highly expressed in the nucleus, a large distribution of Shh was seen in the cytoplasm (Figure 5K).

### 3.4. Shh secreted by senescent renal tubular epithelial cells promoted the activation and proliferation of fibroblasts

**FIGURE 6:**
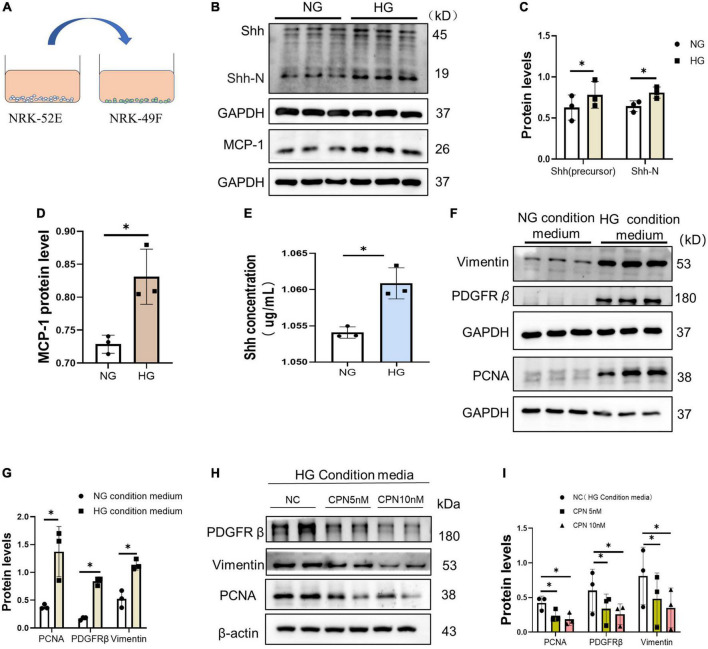
Activation and proliferation of fibroblasts stimulated by HG condition medium. **(A)** Schematic diagram of NRK-49F cell culture with supernatant of condition medium. **(B–D)** Western blot analyses showed Sonic hedgehog (Shh) and p21 protein in each group. Representative Western blot **(B)** and quantitative data **(C,D)** are presented. **(E)** Shh was detected by enzyme-linked immunosorbent assay (ELISA) in condition medium of each group. **(F,G)** NRK-49F cells were cultured with condition medium for 72 h. Western blot analysis showed Vimentin, proliferating cell nuclear antigen (PCNA), and PDGFR-β protein in each group. Representative Western blot **(F)** and quantitative data **(G)** are presented. **(H,I)** NRK-49F cells cultured in the condition medium with or without CPN (5 nM, 10 nM). Western blot analysis showed Vimentin, PCNA, and PDGFR-β protein in each group. Representative Western blot **(H)** and quantitative data **(I)** are presented. All data are presented as mean ± standard deviation (SD) from three independent experiments. *n* = 3; *P < 0 05 vs. NC group.

Next, we conducted a cell condition medium experiment. NRK-49F cells were cultured in the supernatant of NRK-52E stimulated by HG or HG medium for 72 h, and the protein was collected and detected (Figure 6A). The protein levels of MCP-1 and Shh in NRK-52E cells in the HG group were increased (Figures 6B–D). The results of ELISA indicated that the content of Shh in the supernatant of the HG group medium increased (Figure 6E). Furthermore, we detected that the levels of activation and proliferation-associated protein were increased in NRK49F cells cultured in condition medium. The protein levels of Vimentin, PDGFR-β, and PCNA increased in NRK-49F cells in the HG condition medium (Figures 6F, G). To verify the role of Shh, cyclopamine (CPN), an inhibitor of the Shh pathway, was added to HG condition medium. The results showed that the activation and proliferation of NRK-49F cells induced by HG condition medium was diminished after co-culture with CPN. The above results suggested that fibroblasts could respond to HG-mediated Shh secretion by renal tubular epithelial cells (Figures 6H, I).

## 4. Discussion

A variety of pathogenic factors, such as genetic factors, senescence, autophagy, and metabolic disorders, are involved in the progression of DKD. For the past few years, many studies have revealed a correlation between renal fibrosis and senescence. Renal fibrosis is accompanied by renal senescence (28). The phenomenon of cellular senescence is discovered by Hayflick and Moorhead, and was defined as the phenomenon of limited proliferation of cultured human fibroblasts. Replicative senescence in cellular senescence is mainly caused by telomere dysfunction and critical shortening (29). Accumulating evidence put forth that many factors contribute to senescence, and thus the understanding and definition of senescence has been expanding and evolving. Permanent cell cycle arrest caused by stress factors, such as DNA damage and oxidative stress, is known as stress-induced premature senescence (30).

Satriano et al. (31) demonstrated that renal tubular epithelial cells suffer from oxidative stress due to the stimulation of HG and other factors in DKD, and the transformation of cells to senescent phenotype is a major step in the pathogenesis of DKD. Stress-induced cell senescence is a critical mechanism for promoting fibrosis. Stress-induced senescence and EMT promotes further development of chronic kidney disease (CKD) (28). However, the cellular events involved in stress senescence in DKD are not yet fully understood. Some studies (32) have shown that in different renal cells, the expression of p16^*INK*4*A*^ protein increases with age, which is obvious in renal tubular cells. The p16^*INK*4*A*^ is a major inhibitor of cyclin-dependent kinase (CDK), which blocks the cell cycle through the G1 phase by inhibiting CDK4 and CDK6 (31, 33). The p53-p21-RB and p16*^INK^*-RB signaling pathways are responsible for regulating the cell cycle. Activating these two pathways could increase the expression of p21 and p16^*INK*4*A*^, which in turn accelerates the process of cell senescence (34). In addition, cell division into an irreversible state of cell cycle retardation is the most obvious and crucial feature of cell senescence. Therefore, cell cycle arrest occurs, and cell division stagnates in G1 phase in senescent cells. Previous studies suggested that when the cell size increases, the cell cycle process is decreased, and pH-dependent β-galactosidase activity is increased during senescence (35). The deletion of Lamin B1 in senescent cells is a new marker of cellular senescence. In summary, SA-β*-*Gal activity, p16^*INK*4*A*^ protein, and p21 protein expression and the secretion of a variety of bioactive factors increased (senescence-associated secretory phenotype or SASP), while the expression of nuclear membrane protein Lamin B1 decreased during senescence.

Our findings are consistent with those of previous studies that cellular senescence occurs during DKD progression. The high levels of BG, MUA, UACR, SCr, BUN, T-CHO, and TG indicated that *db/db* mice developed disorders of glucolipid metabolism and impaired renal function, accompanied by pathological changes in glomerular hypertrophy and segmental atrophy of the tubules with increased interstitial inflammatory cells (Figure 1). Also, SASP expression was increased in *db/db* mice (Figure 2). Lin et al. revealed that renal tubular epithelial cells in DM undergo senescent phenotype transformation, and high concentration of glucose induced cell senescence (36). We found similar results for NRK-52E cells cultured with HG medium *in vitro*. Furthermore, the cell cycle stagnation occurred under the condition of HG, suggesting that it induced senescence of renal tubular epithelial cells.

Senescent cells acquire SASP and secrete cytokines, such as MCP-1. SASP can destroy normal tissue structure and induce the inflammatory cell infiltration and EMT process (37). It has been shown (38) that the development of inflammation is also a part of the pathogenesis of DKD. The persistent hyperglycemia state activates the inflammatory response through pathways, such as glycosylation end products (ACE), mediates the synthesis and secretion of pro-inflammatory factors, including TNF-α, intercellular adhesion molecule 1 (ICAM-1), IL-6, and MCP-1, and contributes to glomerular basement membrane thickening and fibrous proliferation of small renal arteries. This in turn, causes glomerulosclerosis and tubular atrophy and interstitial fibrosis. Our data demonstrated that HG enhanced the senescence of renal tubular epithelial cells that SASP and promoted infiltration of inflammatory cells and deposition of ECM in the renal tubular interstitium (Figure 3).

The most interesting finding of this study is to confirm the key role of Shh secreted by senescent cells in mediating fibroblast proliferation and renal fibrosis *in vivo* and *in vitro*. The activation and proliferation of interstitial fibroblasts are also the main pathological features of DKD. Myofibroblast is the largest source of ECM in fibrotic tissue (39). Interestingly, the increased expression of Shh promotes the activation and proliferation of interstitial fibroblasts (40). Shh belongs to the hedgehog (HH) family that can be divided into three subgroups in vertebrates, namely, Desert hedgehog factor (DHH), Indian hedgehog factor (IHH), and Shh factor (40). HH signals directly or indirectly control the target genes related to cell proliferation and tissue dynamic balance. For example, HH signal induces cell proliferation by upregulating cyclin D/E and transcription factor FOXM1 (41). *Shh* is widely expressed in human tissue and is a key gene in embryogenesis, tissue homeostasis, injury repair, and fibrosis (41). In this study, the protein levels of Shh precursor and Shh-N peptide increased in the kidney tissue of *db/db* mice as well as *in vitro*. This phenomenon was further verified in the *in vitro* D-gal-induced senescence of renal tubular epithelial cells (Figures 3, 5). The results of immunofluorescence staining showed increased expression of Shh in renal tubular epithelial cells with reduced Lamin B1-positive staining. At the same time, increased the expression of γH2A.X and Shh in the cells, and in cells where γH2A.X highly expressed in the nucleus, a large distribution of Shh was seen in the cytoplasm (Figures 4, 5). Thus, it could be inferred that Shh is secreted by senescent renal tubular epithelial cells and plays a role in the interstitium.

Next, we explored the association between HG-induced senescent renal tubular epithelial cells and Shh. We found that the content of Shh in the supernatant of the HG group medium increased, and the levels of activation and proliferation-associated protein were increased in NRK49F cells cultured in HG condition medium, suggesting that Shh secreted by senescent cells activated fibroblast proliferation (Figure 6). Consistently, the inhibition of Shh signaling by CPN could selectively block the proliferation of fibroblasts (Figure 6). Zhou et al. (39, 42) showed that in different types of CKD, Shh specifically upregulated in renal tubular epithelial cells. It activated mesenchymal fibroblasts and promoted their proliferation through various mechanisms. Shh also promoted the expression of FN and Vimentin and mediated the deposition of ECM (39). In ischaemia reperfusion injury (IRI) mouse model (39, 42), overexpression of Shh promoted the proliferation of fibroblasts and ECM overproduction and deposition, eventually leading to kidney fibrosis and aggravated kidney function damage. Fabian et al. demonstrated that recombinant Shh protein activated Ptch1/Gli1 signaling pathways in fibroblasts and induced the expression of α-smooth muscle actin (α-SMA), FN, and type I collagen, suggesting that Shh signal transduction activated fibroblasts to synthesize ECM (43). Taken together, our results provide a new finding that senescent renal tubular epithelial cells secrete Shh. Therefore, we concluded that HG-induced secretion of Shh by senescent renal tubular epithelial cells promotes the activation and proliferation of tubulointerstitial fibroblasts to aggravate tubulointerstitial fibrosis.

In conclusion, HG induces renal tubular epithelial cell senescence, its secretion of senescence-associated proteins and Shh mediates inflammatory responses and fibroblast activation and proliferation, ultimately leading to renal fibrosis.

## Data availability statement

The original contributions presented in this study are included in the article/supplementary material, further inquiries can be directed to the corresponding authors.

## Ethics statement

The animal study was reviewed and approved by the Institutional Animal Ethics Committee of Guizhou Medical University.

## Author contributions

DW completed the experiments and wrote the manuscript. LY completed the data collection and performed the statistical analysis. RC, LL, JX, XZ, HZ, WT, and HD guided the experimental methods. YW and BG conceived and revised the manuscript and approved the final edition of the manuscript to be published. All authors contributed to the article and approved the submitted version.
